# Analysis of UAV-Acquired Wetland Orthomosaics Using GIS, Computer Vision, Computational Topology and Deep Learning

**DOI:** 10.3390/s21020471

**Published:** 2021-01-11

**Authors:** Sarah Kentsch, Mariano Cabezas, Luca Tomhave, Jens Groß, Benjamin Burkhard, Maximo Larry Lopez Caceres, Katsushi Waki, Yago Diez

**Affiliations:** 1 Faculty of Agriculture, Yamagata University, Tsuruoka 997-8555, Japan; larry@tds1.tr.yamagata-u.ac.jp; 2 Faculty of Natural Sciences, Leibniz Universität, 30167 Hannover, Germany; luca.tomhave@kabelmail.de (L.T.); gross@phygeo.uni-hannover.de (J.G.); burkhard@phygeo.uni-hannover.de (B.B.); 3 Brain and Mind Centre, University of Sydney, Sydney 2015, Australia; mariano.cabezas@sydney.edu.au; 4 Faculty of Science, Yamagata University, Yamagata 990-8560, Japan; waki@sci.kj.yamagata-u.ac.jp

**Keywords:** ArcGIS, big data, blueberries, deep learning, image analysis, orthomosaics, segmentation refinement, UAVs

## Abstract

Invasive blueberry species endanger the sensitive environment of wetlands and protection laws call for management measures. Therefore, methods are needed to identify blueberry bushes, locate them, and characterise their distribution and properties with a minimum of disturbance. UAVs (Unmanned Aerial Vehicles) and image analysis have become important tools for classification and detection approaches. In this study, techniques, such as GIS (Geographical Information Systems) and deep learning, were combined in order to detect invasive blueberry species in wetland environments. Images that were collected by UAV were used to produce orthomosaics, which were analysed to produce maps of blueberry location, distribution, and spread in each study site, as well as bush height and area information. Deep learning networks were used with transfer learning and unfrozen weights in order to automatically detect blueberry bushes reaching True Positive Values (TPV) of 93.83% and an Overall Accuracy (OA) of 98.83%. A refinement of the result masks reached a Dice of 0.624. This study provides an efficient and effective methodology to study wetlands while using different techniques.

## 1. Introduction

Recent changes in global climate conditions influence species composition and accelerating the presence of invasive plant species in natural environments. Species that spread outside their native habitat and rapidly and effectively adapt to new environments are known as invasive species [1]. The spread of invasive species often benefits from ecosystem changes and habitat disturbances that weaken the natural species and open an ecological niche for invaders. Hence, invasive species can influence the biodiversity, thus limiting the growth of natural plant species due to a higher occurrence of an invasive species, which could lead to ecosystem degradation [2]. The fast adaption to multiple stress factors in environments could also lead to a replacement of native species and it may increase economic costs due to production losses in agriculture and forestry [3]. In Europe, 11% of the 12,000 identified species have caused damage to the economy, society, and the environment [4]. Reference [5] states that hundreds of invasive species find their pathways through horticulture, agriculture, etc., and the linearly increasing trend of invasive species numbers (from 1970 to 2007) indicates higher impacts of invasive species in the future. Reference [6] pointed out that not only invasive species have an impact on native plants, since several factors often interact with the environment that influence species distributions. In recent years, the need to precisely understand the ecological impacts of invasive species in ecosystems has become a key in designing and prioritizing natural resource management approaches [2], since the behaviour and impact of invasive species is still not well understood [3,7]. Furthermore, a high number of invasive plant are species spreading in natural environments, which increases the demand of management practices [5].

In the past two decades, an explosive spread of North American blueberry hybrids (*Vaccinium corymbosum* x *angustifolium*) has been observed in several moors in the northern German geest area, endangering the natural development of these protected raised bog areas [8]. The starting point of the spread has been almost exclusively located at existing or former commercial blueberry plantations [9,10], which can be found near or in the immediate vicinity of bogs or former peat extraction areas, due to good soil and local climatic conditions. Most of the recipient habitats are pine forests and bogs in various stages of de- and regeneration. Because of these characteristics, the American Blueberry (*Vaccinium angustifolium* x *corymbosum*) has been classified as a potentially invasive neophyte by the *German Federal Agency for Nature Conservation* [11]. After the degradation of wetland areas due to anthropogenic activities, protection programs, called "Moorschutzprogramme", were established by the state government of Lower Saxony for the conservation and the development of rare animal and plant communities in these areas [12]. Furthermore, activities that could threaten the goals of the protection program are prohibited, which increases the difficulty of conducting relevant field studies [12]. However, maintenance and development measures are needed in order to rehabilitate protected and relatively sensitive wetland areas, into which the invasive blueberry species *Vaccinium corymbosum* x angustifolium has migrated. In 2011, 20 counties in Lower Saxony reported stands of spontaneously growing blueberry bushes [13]. The potential area that is occupied by spontaneously growing blueberry bushes can reach several square kilometres within a few years [14]. Previous studies, as presented in [10,14], used grids in the field in order to plot the distribution of blueberry bushes within wetlands. Both of the studies focused on sites near blueberry cultivation areas, as the biggest spread was found in close proximity to blueberry plantations [9]. Those studies show the limitations in the studied area and lack an overview. It is still unclear how far the blueberry bushes have already spread and in which areas they occur. In order to implement effective measures in these areas and minimise the disturbance of sensitive biotopes, it is necessary to locate the individual blueberry bushes as accurately and early as possible. In addition, the following questions arise: does a displacement of natural species occur and where should what measures be taken against a continuing invasion? According to [14,15], relatively simple counter measures can lead to good results and prevent further spread, especially when invasive blueberry bushes are identified early. Therefore, a suitable and non-invasive method for recording stock development and distribution is needed. A simple tool is needed to rapidly, cost-effectively, and precisely detect invasive species in wetlands to counteract their rapid reproduction. Wetlands are protected environments with limited ground accessibility making UAVs particularly appropriate for data collection. UAVs offer the possibility to cover large areas with high resolution images, and they have proved their usefulness in a variety of studies in agriculture [16,17] and forestry [18,19,20]. Still, UAV images present challenges like the pre-pocessing of the data. Large amounts of data need to be processed, labeled, and annotated by experts, which is usually time consuming, before the data can be further analysed. The use of deep-learning techniques reduces the amount of time that is needed to extract information from the data, which increases the benefits for several applications.

Images that are acquired by UAVs can be analysed while using computer vision and GIS techniques. Important results can then be obtained by reducing the complexity contained in the images (using different image interpretation strategies) and the findings can be presented in elaborate visualisations [21]. Persistent homology, a tool of topological data analysis, can help to understand complex datasets by analysing their large scale geometric features [22]. In our study, we have used persistent homology to measure the spread of invasive species. Processing images and creating orthomosaics allow for a fast analysis of large amounts of data. Deep learning techniques additionally automatize classification and localisation processes, and make it possible to incorporate expert knowledge into the automatic image processing pipeline. This has the potential to increase the scale of the resulting studies to reach large regions that are significant in terms of country-level invasive species detection and management.

In this study, the incorporation of all the mentioned techniques from remote sensing, GIS, computer vision, computational topology, and artificial intelligence allows for us to study invasive species on a large scale, with minimum disturbances and the incorporation of expert knowledge. To the best of our knowledge, this is the first study that includes different techniques and UAV gathered data to increase the understanding of an invasive blueberry species in wetlands. Furthermore, locating and studying small bushes in large areas and at the single-bush level was done for the first time. Therefore, UAV-supported methods offer an efficient possibility to discover individual young plants on a large scale and detect propagation hotspots at an early stage.

The following objectives guided this study:

(I) To use UAV data in order to provide allometric statistics (height, area, and number) of invasive blueberry in large areas at bush level.
(II) To use clustering techniques and persistent homology to quantitatively and qualitatively assess the spread of blueberry invasions.
(III) To assess the potential of Deep learning to automatically segment blueberry bushes, initiating the possibility for even larger-scale studies.

### State of the Art

In recent years, remote sensing techniques have been used in various natural environments with the goal of reducing the need for in situ measurements [23]. Low-cost data gathering, time saving, and larger study areas are the benefits. Furthermore, data can be directly used and processed within Geographical Information Systems (GIS) [23]. This has been done successfully, for environmental studies [24,25,26]. Closely related to the current work, Reference [27] proposed using GIS as a synthesising tool in invasive species management approaches. Reference [28] used satellite images of 1992 and 2002 in order to identify stress indicators and change detection in a wetland in Sri Lanka to quantify the conditions of the complex. The authors state that the inventory, mapping, and monitoring are needed to understand interactions in the ecosystem. Classification with the Maximum Likelihood Algorithm were performed, mapping and spatial analysis were used, and finally refined and verified with ground data. Their approach ([28]) reached 86% accuracy and provided detailed analysis. GIS in combination with remote sensing data was found to be an effective methodology for investigating wetlands. Reference [29] evaluated vegetation change detection while using the NDVI of remote sensing data and applied GIS in order to visualise the results. Landsat and Shuttle Radar Topography Missions were used to capture the Vellore District. Image interpretations were carried out using ERGDAS IMAGINE software in order to classify and detect changes in the vegetation. The differences in the NDVI values were used to analyse data sets of the years 2001 to 2006. The study provided information about the lowest decrease in the forest area by 6%, while agriculture land increased the most by 19%.

In comparison to satellite images UAV images provide a higher resolution and appear to be more suitable for wetland investigations, especially when focusing on invasive species. Higher resolution images allow for higher accuracies of image interpretations and feature extractions [30]. Several studies using UAVs in wetlands have already been carried out [31,32,33,34]. Reference [34] developed a method for detecting and mapping invasive species with UAVs. The authors acknowledged UAVs as suitable for monitoring eradication efforts in wetlands. Reference [33] realized the higher efficiency in gathering valuable and accurate information in comparison to field studies, when using UAVs and computer vision techniques to enhance classifications and health assessments in wetlands. Gandhi et al. [31] used UAV imagery for detecting invasive species and mapping their distribution and spread. This study also compared data from two years (2009 and 2011) and detected an increase of 19.07%, which was confirmed by field studies with a total agreement of 94% and shows the suitability of UAV imaging for this kind of application. Reference [31] lanalysed the spread of *Spartina alterniflora* in Beihai in the years 2009 and 2011, using high resolution images acquired by UAVs. They captured images at a flight height of 800 m, generated orthomosiacs, performed multi-resolution segmentation by grouping homogenous pixels, and classified them. The target species was extracted by their pixel values. In a final step the accuracy was assessed and verified with field data by comparing three sample plots (a total of 166 samples) with the image results. A total accuracy of 94.0% could be achieved and, hence, provided information regarding an increasing spread of 19.07% from 2009 to 2011. The total infected area was, in 2011, 357.2 ha. Moreover, the image analysis provided the opportunity to identify areas with different levels of densities. Reference [35] collected UAV images of *Harrisia pomanensis* in the Limpopo province of South Africa. An area of 87 ha was captured by images that were taken at a height of 800 to 817 m. Orthomosaics generated with Agisoft Photoscan and pixel as well as object-based classifiers were used. The classification results of supervised and unsupervised classifiers were assessed. The supervised classification outperformed the unclassified one, and the object-based approach outperformed the pixel-based one. The best accuracy achieved was 86.1%.

Given the sizes of the data sets used and the need to detect and classify parts of the images that only trained experts can confidently tell apart the use of deep learning in problems that are closely related to the current work is of common use. For example, a recent study [36] used CNN (convolutional neutral network) in order to classify images from wetlands in an area of 700 km${}^{2}$. They used a fully trained and fine-tuned CNN with a limited amount of data. A comparison of the CNN with random forest was performed to evaluate the capacity and classification accuracies of CNNs. Canadian wetlands were captured with two RapidEye images with 5 m resolution in 2015. The validation data were sampled in 2015 and 2016 and four wetland classes were identified using 1000 samples. The network was trained with patches and 30,000 iterations and then tested in a second step. The CNN outperformed the random forests and an overall accuracy of 94.82% was achieved, varying between 76.65% and 98.74%. Deep convolutional neural networks were also used by [37] in order to classify AUV-acquired wetland images. The 677 m × 518 m study area was located in Southern Florida. The authors used processed orthomosaics and multi-view images and then compared them with the performances of random forests and support vector machines. Image segmentation was done with Trimble’s eCognition by first segmenting objects, then extracting features, and finally training a classifier. The results of the study show the advantages of deep CNN, reaching an accuracy of 82.02%, when multi-view images were used and with lower accuracy when orthomosaics were used (71.69%). A similar approach was used in [38]. 3800 images of the Brazilian national forest (Kaggle dataset) were used in order to identify *hydrangea* in the images. The dataset contained two-thirds of images, including the invasive species, a smaller fraction, where the invasive species appeared only in parts of the images, and a third small fraction that included no plants at all. The authors used three models: VGGNet, DenseNet, and Inception, which were pre-trained with ImageNet. Data augmentation was used and accuracies of 97.6% were reached.

## 2. Materials and Methods

### 2.1. Study Area

**Figure 1:**
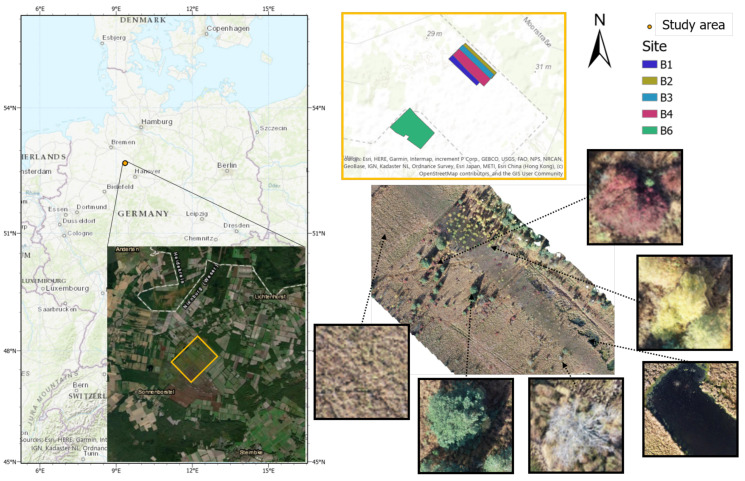
Location of the study area in the north of Germany. In the upper right part of the figure, the study sites are marked with different colours. The bottom right part an example orthomosaic is shown with detail of the different classes.

The study area “Lichtenmoor” is located in a wetland region about 60 km northwest of Hanover in Lower Saxony, Germany (52°43${}^{\prime }$06.2${}^{\prime \prime }$ N 9°20${}^{\prime }$41.5${}^{\prime \prime }$ E) (Figure 1).The total size of the moor area is 38 km${}^{2}$ [39]. Post industrial peat cutting characterises the central area of the studied wetland. As a subsequent use, some former peat cutting areas have been rewetted with the aim of regenerating raised bogs. In the surrounding area, parts of the Lichtenmoor have been designated as nature reserves. Former hand peat cutting sites are located at the edges and in parts of the nature reserves. Agricultural areas dominate the remaining areas, mostly grassland, dry to moist moorland forests, scrubby heather and moorland degeneration stages, pioneer stages of moorland rewetting, and peat extraction areas under cultivation. The case study area covers a total area of 62 ha and it is located in the central part of the Lichtenmoor region. To the northeast, it borders a pine plantation, while at its southwest border current peat extraction areas are located. Blueberry plantations can be found throughout the Lichtenmoor, especially on the outskirts of the localities Lichtenhorst, Heemsen, Sonnenborstel, and Steimbke.

### 2.2. Characteristics of the Blueberry Species

Blueberries have been cultivated in commercial plantations in Lower Saxony since the early 1930’s [14]. Since then, the area under cultivation has steadily increased. In 2005, the area with blueberry cultivation in Lower Saxony was approximately 1400 hectares [13]. In nature, blueberries are mainly distributed by birds and small mammals, who spread the seeds. Once established, plants can spread in a vicinity by clonal growth and the high regeneration potential of blueberries favours a strong spread [10]. The species prefers acidic locations, such as pine forests or wetlands. Especially, raised bogs in their degeneration stage provide ideal habitat conditions for invasive blueberries [14]. Thus, blueberries are growing increasingly wild in neighbouring areas. References [9,14] found a correlation between the density of blueberries and the distance to blueberry plantations; a maximum distance of 1700 m was recorded. Near cultivated areas, the feral blueberries form dense shrub stands with height of up to 2–3 m and have a ground coverage of up to >60%. With increasing distance, the degree of coverage decreases rapidly [8,9,13]. Since these studies were already conducted in the last millennium, a larger distribution must be expected today. Reference [10] describes a distribution of blueberry bushes over 4 ha in the “Krähenmoor”, where the maximum distance to the plantation was identified at 100 m. Blueberry bushes are low and can reach a height of 60 cm, but, occasionally, tall species can be found with a height of 3 m [10]. In the course of the increasing growth and dense shrub structures, other ground vegetation is displaced, since it cannot exist under the shade of blueberries. Other presumed effects of blueberry cultivation are reduced evaporation rates and the influence on nutrient cycles in wetlands, which, in turn, can have an impact on existing plant species. Therefore, human interventions are necessary to protect the sensitive rare structures and characteristic plants of wetlands [10].

### 2.3. Data Collection and Pre-Processing

**Figure 2:**
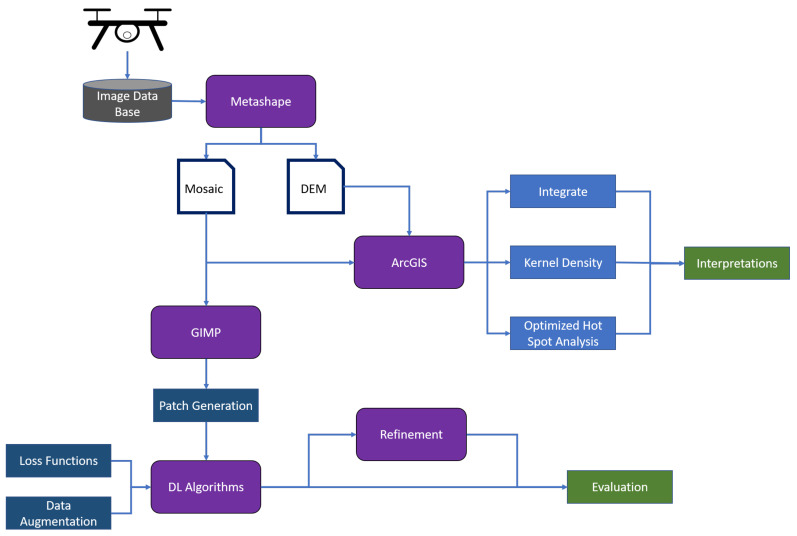
Workflow of the paper. Gray is our data base; purple boxes show softwares/programs used in this study; white boxes with a blue outline are generated files; dark blue are processes used for the deep learning (DL) classification and detection; light blue are tools in ArcGIS.

Image collection was performed by using a DJI phantom 4 UAV in autumn 2018, because of the seasonal red colouring of blueberry leaves, which makes them easily detectable (Figure 2). For sites B1 to B4, 490 to 584 images were collected, while 1346 images were taken for B6. The flight height was 50 m and front and side overlaps of 80% were chosen. These images were then processed while using the Metashape software [40] to align images in order to produce one orthomosaic and DEM (Digital Elevation Model) for each site (Figure 1). It should be mentioned that an overlap between the sites B1 to B4 was chosen, so the east and west borders of the orthomosaics B1 to B4 are overlapping. All of the obtained orthomosaics were annotated while using the open source image manipulation software GIMP [41]. For three of them (B1, B2, and B3), the whole orthomosaic was annotated and each pixel was given one of the following six labels: blueberries, trees, yellow bushes, soil, water, and dead trees (Figure 1). The class trees contains pine trees (*Pinus sylvestris*), the class yellow bushes contains shrubby birches (predominantly *Betula pubescens*, secondarily *Betula pendula*). Binary layers for each of the six classes were created for each of the three orthomosaics while using the pixel-level labels. These annotations were based on colour, shape, and context information contained in the orthomosaics. In the last two orthomosaics (B4 and B6), only blueberry bushes were annotated.

In order to train deep learning models to detect blueberry bushes orthomosaics annotated with all of the aforementioned labels were needed. Therefore, only B1 to B3 were used in the deep learning section of this study. These three orthomosaics, as well as the corresponding annotated binary layers were divided into axis-parallel patches of side length = 100 (hereafter "patch size"). This value was determined by taking the sizes of the blueberry bushes in the images, which ranged from 20 to 100 pixels in radius, into account. The classes in each patch were stored in a separate *label* list. In general, patches contained more than one class and, therefore, our problem can be defined as a multi-label patch classification problem.

#### 2.3.1. Data Processing Using Arcgis

This section deals with data processing performed with ArcGIS pro 2.4.1 and python in order to identify parts of the orthomosaic containing blueberry bushes, in order to visualize them and analyse their characteristics. ArcGIS is a Geographic Information System software that visualises and comprehends geographic data. The software provides over 1000 tools to analyse real world data, including UAV-acquired images. The mapping options allow to visualise the gathered data within the correct location in an eligible base map. The primary purpose of this study was to provide information about the location and distribution of invasive blueberry species and map them for management.

In this context, the positions of the blueberry bushes in the five orthomosaics were digitised by hand as point data in an ArcGIS shapefile and several analytical tools were then used (Figure 2). The *kernel density* tool functions were used to calculate the magnitude-per-unit area from the blueberry points. Smaller search radiuses were used to show a detailed density raster. The tool *integrate* was used to group blueberry bushes that fall into a specified distance, as specified distance, 3 and 6 m were used. With the tool *collect event*, the number of points which were integrated before, were summarized in a new layer. Those steps were necessary to perform an *optimized hotspot analysis* of the blueberry abundance with the blueberry point shapefile. The hotspot analysis identifies the significant difference between the neighbourhood of a feature in comparison to the extent of the respective study area. Is the value of a feature significantly higher, it is considered to be a hotspot and the tool provides a feature map with three levels of confidence (90%, 95%, and 99%). As input for the hot spot analysis, the created layer of the tool *collect events* was used and analysis field counts were chosen. Furthermore, it also indicates the significantly lower features.

On the basis of the annotation made for all orthomosaics, several simple python codes and ArcGIS were used to perform image analysis. Pixels were counted for all orthomosaics, as well as all annotated layers. The number of black pixels in the annotated layers were specifically counted in order to obtain the percentage and area in m${}^{2}$ per class. Additionally, the overall area presented in the orthomosaics was calculated in hectares. Because the focus of this study was on the blueberry bushes, several statistical values were generated: the number of blueberry bushes was counted and the number of blueberry bushes per ha was calculated for each orthomosaic. Additionally, the total area, as well as the area per blueberry bush, were computed in m${}^{2}$. Furthermore, the proportion of blueberry bushes in relation to the vegetation was calculated in %. Finally, height values were computed on the basis of DEMs, annotations of the blueberry bushes, and annotations of ground points per site. Ground points were annotated close to blueberry bushes in order to increase the accuracy of the computed height. Maximum height values were estimated in a first analysis and the median height in a second analysis to evaluate the results.

### 2.4. Persistent Homology

Persistent homology provides topological information of complex datasets [42] at different spatial resolutions. This information deals with the connectivity of nearby points and it can be computed in different dimensions. For the purpose of this study, we worked with 0-D persistent homology (usually expressed in the form of H0 diagrams). H0 homology can be seen as growing disk at uniform radius-increase speed around pre-defined data points. In order to use this tool, we first discredited the manually annotated blueberry regions by uniformly sampling them. Subsequently, the radius around each sample point was increased to grow blueberry bush regions. When two blueberry regions were merged because of the expansion, one of the regions was considered to be dead because it was absorbed by the other region. As time passed, the number of connected blueberry regions decreased, and finally all of the regions were connected in one region.

The H0 diagram shows the change in the connectivity of the blueberry bushes by plotting the time/radius when blueberry regions get connected. False regions that were produced by our sampling of the annotated regions were discarded and only those parts of the diagrams that were obtained after all sampling points in each region had been joined together were considered. Based on this outcome, the radius was calculated until 1%, 10%, 50%, and 90% of the blueberry regions were connected and plotted. The diagram will vary greatly, depending on the number and position of blueberry bushes in the input image.

### 2.5. Deep Learning Techniques

Deep learning is a trending field of machine learning that focuses on fitting large models with millions of parameters for a variety of tasks, such as image classification and segmentation. These approaches have been rapidly gaining attention in computer vision tasks, due to their recently increased accuracy. Deep learning models commonly learn from examples in a supervised manner. First, an *architecture* or a graph of connected nodes is defined. These nodes are often grouped in *layers* that perform a specific operation, and the combination of a large number of layers is referred to as a *deep neural network* (**DNN**).The typology of the nodes, the number of nodes per layer and the connections between them determine the behaviour of the network. In general, two main types of nodes are used: linear nodes, expressed as matrix multiplications and nodes that introduce non-linear functions (such as the sigmoid function). The weights in linear nodes are usually initialised with random values following a specific distribution. Afterwards, the network is given samples of the data, known as *training samples*, which contain instances of the problem (i.e., image intensities) with their corresponding solutions (i.e., labels). These samples are iteratively run through the network in order to evaluate its current accuracy and the weights are updated following an optimization process.

In this study, DNNs were used to locate and identify the six classes that are defined in Section 2.3, with a focus set on the blueberry class. The basis for this deep learning approach is described in previous studies [20,43], which led to the use of the algorithms that are described in this section.

Our approach is based on a patch classification model that uses the patches of 100 × 100 pixels described in Section 2.3. A patch of orthomosaic B1 and B3 covered; therefore, 700 cm × 700 cm and a patch of orthomosaic B2 500 cm × 500 cm. For each patch, a list containing the class labels was created from the binary maps for each class. This classification is usually referred as multi-label, since each input patch might contain different labels (i.e., a part of the patch may contain soil, while other parts of the same patch could also contain bushes or blueberries).

Deep neural networks for classification have two major components: a feature extraction stage and a prediction stage. At the first stage, convolutional operators are trained in order to extract salient and meaningful features (such as texture) while at the second stage these features are used to predict the final labels for the given input patch or image. In order to train general and robust feature extractors, a large pool of heterogeneous images with different properties (lightning, colour, view, etc.) is needed to capture all of the possible image variabilities. However, as proven by our previous work [20,43], transfer learning is a useful tool for image analysis applications, where the training dataset is too small to properly train these feature extractors from scratch.

In our case, only three different orthomosaics are available; hence, we decided to use transfer learning by loading a pre-trained ResNet50 architecture with weights from ImageNet, due to its accuracy and reduced training time. ImageNet is one of the largest image databases for image classification research, with more than 80,000 labels and at least 1000 images for each label.

In order to perform the evaluation of the proposed model, two of the three orthomosaics were used for training and validation and the third one was used for testing. This cross-validation strategy is usually referred to as a leave-one-out strategy. All of the orthomosaics were used once for testing, training, and validation by rotating them for each experiment. Patches from the testing orthomosaic were not included for training or validation to avoid data leakage during training.

Two main approaches were used in order to obtain a higher detection rate for the blueberry class:

- Data augmentation is a commonly used strategy in deep learning and it can increase the size of the training datasets without the need to collect new data. In this case, data augmentation was used to generate new synthetic patches of the blueberry class, which was the less frequent class (see Section 3.2 for details). Six image transformations to augment the data were used: up/down and left/right flips; small central rotations with a random angle, in order to simulate different perspectives of the bushes; Gaussian blurring of the images, which simulates blurring due to the movement of the UAV; linear and small contrast changes, which can represent different light and shadow conditions and localised elastic deformations. In order to implement this transformations we used the “imgaug” library [44].
- Loss functions are used to compute the accuracy of the network and update their parameters. By giving different weights to different classes, their importance can be changed during training. In this study, two loss functions were used; the first function checks if a patch contains a blueberry or not, while the second one checks the fraction of blueberry pixels inside the patch. The optimal training settings followed the ones that were used in our previous study [43].

We considered labels for all the patches used and the relation between (1) predicted values resulting from our algorithm and (2) real values as stated in the manually-annotated ground truth in order to assess the predictive power of our algorithms.

We then broke all of the patches into the usual classification categories of:

- **True Positives** or TP, predicted to contain the blueberry class and also marked in the ground truth as containing them.
- **False Positives** or FP, predicted to contain the blueberry class but NOT marked as such in the ground truth. These patches correspond to over-prediction errors where the algorithm “sees” the blueberry class when it is not really there.
- **True Negatives** or TN, not containing the blueberry class in the prediction or in the ground truth.
- **False Negatives** or FN, not predicted to contain blueberries, but actually being marked as containing them in the ground truth. These patches correspond to under-prediction errors where existing blueberry instances are missed by the algorithm.

We used the two following metrics (True Positive Rate or Sensitivity and Accuracy) in order to summarize the occurrences of the four categories.

(1) $$\begin{array}{c} TPR=SENS=\frac{TP}{TP+FN}\;ACC=\frac{TP+TN}{TP+TN+FP+FN} \end{array}$$

Finally, we modified the algorithm to present the results in a way that was more usable for end-users. The 100 × 100 patches used for prediction managed to capture most of the occurrences of the Blueberry class (see Section 3.2 for details). However, these rather large patches also included large areas that actually contained no blueberries. While expert users could easily use these results as a starting point in order to quickly identify the exact location of blueberry bushes, we felt that refining our prediction using smaller patches would make their work faster, while also providing clearer information for non-expert users. Consequently, we divided each of the predicted 100 × 100 patches into 16 25 × 25 patches, re-sampled each of these newly-made smaller patches to the image size that is used by our DNN and re-classified them. This resulted in a refined result made up of 25 × 25 patches. This process had the disadvantage that, if errors were made, some of the correctly predicted blueberry pixels might be lost. In order to evaluate this issue, we considered the *TP*, *FP*, *TN*, and *FP* status of each pixel in each patch and measured the percentage of positive pixels that were covered by our predicted patches as well as the Dice coefficient that gave us an indication of the relative weight of blueberry pixels inside of our predicted patches:

(2) $$\begin{array}{c} Dice=\frac{2TP}{2TP+FP+FN} \end{array}$$

## 3. Results

**Figure 3:**
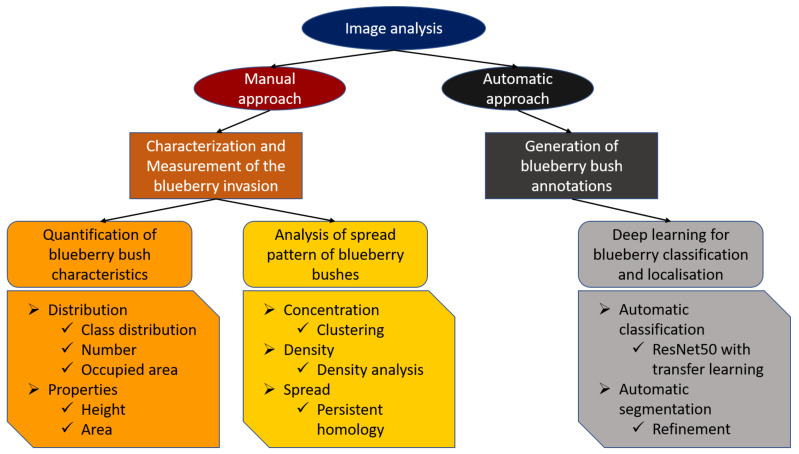
Image analysis workflow. Consists of two parts, the manual approach using manual annotation, GIS and persistent homology, and the automatic approach while using deep learning and segmentation to analyse the blueberry invasion.

This section is divided in two parts: the first part of the analysis focused on the manual annotations of the wetland vegetation and, most specifically, of the blueberry bushes. GIS, computer vision, and persistent homology were used to describe and quantify the characteristics of the blueberry invasion in all of our test sites. In the second part of this section, the results of Deep learning techniques are presented. In this case, our goal was to assess to what extent these technologies can be used to automatically generate the annotations that were used in the first part to characterise the invasion. The general workflow can be seen in Figure 3.

### 3.1. Analysis of the Blueberry Invasion

In this part of the study, we focused on characterising and measuring the blueberry invasion of the wetland.

#### 3.1.1. Quantitative Analysis of Blueberry Bushes

The distribution of the classes in the images (blueberries, trees, yellow bushes, soil, water, and dead trees, see Section 2.3) was analysed and the state of the invasion was assessed by gathering information regarding the areas of the sites, the numbers of blueberry bushes, and the average area per bush.

**Figure 4:**
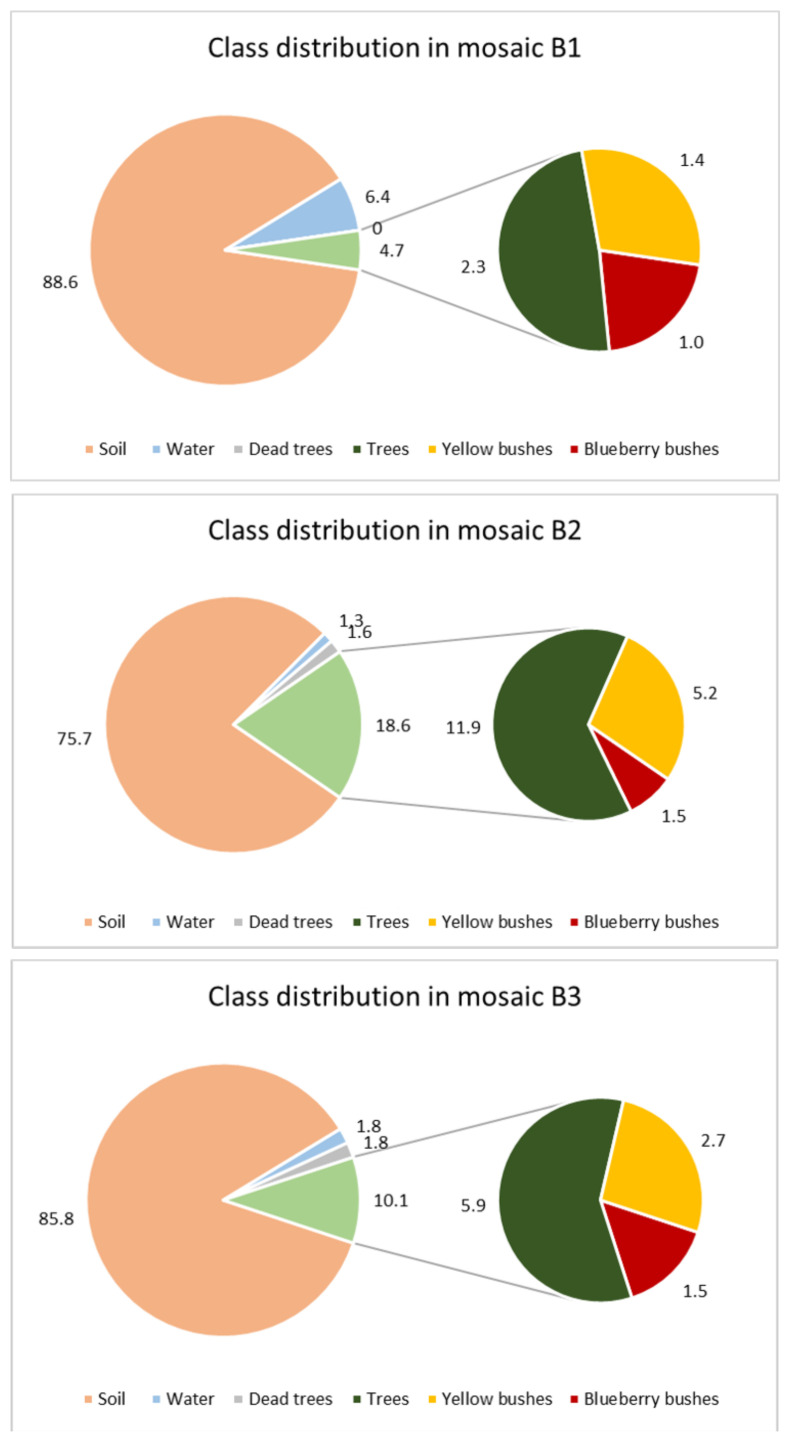
Distribution of annotated classes for the orthomosaics B1 to B3.

One important aspect was to calculate the area of blueberry bushes within the orthomosaics. The area covered by the orthomosaics varied between 10.6 to 12.5 ha, only B6 was larger with 15.5 ha (Table 1). Together with the annotations made for B1 to B3 the area of each class was calculated (Figure 4). As can be seen in the orthomosaic (Figure 1) the main part of the image represented soils. This was validated by the area calculations: with 76% of the orthomosaic B2 and 89% of B3, soil represents the highest values of all classes. The second smaller pie shows the living vegetation varying between 4.7% in B1 to 18.6% in B2. Out of the living vegetation, 8.2% (B2), 15.0% (B3), and 21.1% (B1) are blueberry bushes. In most of the orthomosaics blueberries were the least frequent class (with 1 to 1.5%).

The number of blueberry bushes varied from 235 in orthomosaic B6 to 687 in B2 (Table 1). The site areas of orthomosaic B1, B3 and B4 were similar, while orthomosaic B6 is the largest site containing the least number of blueberry bushes and orthomosaic B2 contains the greatest number of blueberry bushes in the smallest area. The ratio could be confirmed by calculating the blueberry bushes per ha (Table 1). In another step, annotations were used in order to calculate the total area covered by blueberry bushes. In orthomosaic B6, an area of 278.07 m${}^{2}$ was covered by blueberry bushes, which represents the smallest area and it resulted in an area of 1.18 m${}^{2}$ per blueberry bush. The largest area was covered by blueberry bushes in B2 with 1885.51 m${}^{2}$. The average size of the bushes were similar for B2 and B3. In site B1 the average size of blueberry bushes was the highest with 3.55 m${}^{2}$, while the covered area was third lowest with 1331.41 m${}^{2}$.

**Figure 5:**
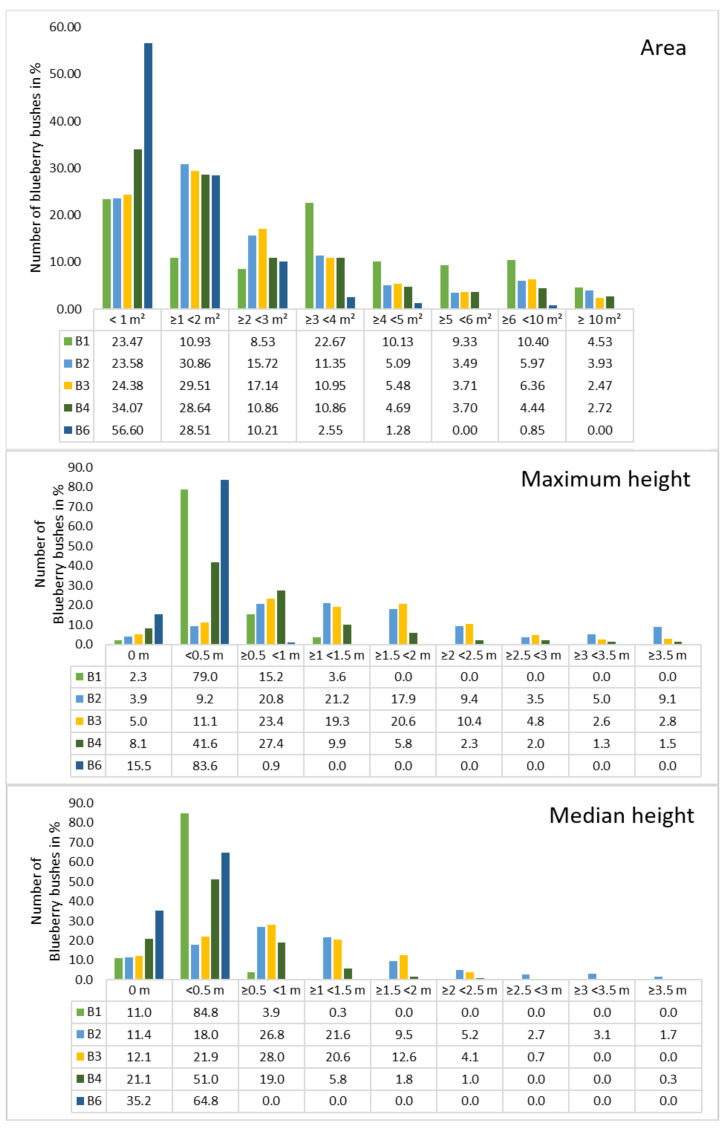
Distribution of the area and height values of blueberry bushes. From top to bottom: area, maximum height, and median height.

Because the covered area and the average size of the bushes could be calculated, the next point of interest was the area and height per blueberry bush (Figure 5). Bushes were grouped into six to smaller than 10 m${}^{2}$ and over 10 m${}^{2}$ because the percentage of blueberries decreased towards larger cover areas. B1 and B2 had approximately 30 bushes between 6 and 8 m${}^{2}$, which was the maximum of all sites. The mean areas were computed for all orthomosaics, indicating that B1 had a high number of large bushes with a mean area of 3.57 m${}^{2}$. The smallest blueberry bushes could be found in orthomosaic B6 indicated a mean value of 1.18 m${}^{2}$. In general, most of the blueberry bushes showed areas of up to 2 m${}^{2}$, a lower amount distributed between 2 and 4 m${}^{2}$ and the lowest numbers distributed in areas greater than 4 m${}^{2}$. B1 was an exception, with around 10% per class over 4 m${}^{2}$. The highest areas calculated range between 17 to 25 m${}^{2}$, with B1 containing four bushes in that range and 27 with areas above 10 m${}^{2}$. The lowest areas were found to be less than 10 cm${}^{2}$ for B1/B4 and approximately 15 cm${}^{2}$ for all other orthomosaics.

A similar distribution can be seen in Figure 5, where the number of blueberry bushes were plotted against the height. Classes were chosen for each 0.5 m starting with 0 m up to lower than 3.5 m and more than 3.5 m. This distribution was chosen due to the characteristics of shallow blueberry bushes that reach 60 cm and tall species that reach 3 m. Regarding the maximum height, no height was computed for 2.3% (B1) up to 15.5% (B6) while the numbers were higher when the median height was considered (11.0% for B1 up to 35.2% for B6). In general, the median height values were higher for the classes 0 m and 0.5 m in comparison to the maximum height, while the values are lower from 0.5 m.The lowest height values started from 0.01 m (B6), 0.03 m (B1), 0.07 m (B3/4), and 0.1 m (B2) for both max. and median height. In general, the maximum and the median height distribution of the orthomosaics was similar. Almost all of the blueberry bushes in orthomosaic B6 were within the class < 0.5 (83.3%). In B1 the number of blueberry bushes in this same class was 79% and 15.2% was between 0.5 and <1 m, which was similar to B6. Orthomosaic B2 to B4 showed a Poisson distribution, whereby B2 had the highest number in 1<1.5 m with 21.2 m, while 41.6 of the blueberry bushes in B4 had the highest value in <0.5 m. Furthermore, in B2 and B3, more than 50% of the blueberry bushes reached heights between 1 m and 3.5 m (57% and 57.7%).It has to be considered that the area was calculated on the shapefiles in ArcGIS, while our developed python code was used in order to calculate the height of the blueberry bushes. Because the input for the code was the annotations of the blueberry bushes that were stored as a PNG file, those bushes that were close together were grouped. Therefore, the height values were not always calculated for an individual bush, which resulted in a different number of blueberry bushes per site: 309 (B1), 519 (B2), 461 (B3), 394 (B4), and 219 (B6).

#### 3.1.2. Analysis of Spread Patterns

In a second step of our quantitative analysis of the blueberry invasion, the concentration, density, and spread patterns were examined. GIS and persistent homology were used to assess these issues. Characterisations of concentrations and densities can indicate the number of blueberry bushes within a given region of the orthomosaic, which exceeds a simple location because the distribution of the bushes can be analysed precisely. Clustering bushes and mapping densities further increase the understanding of the distribution. Together with the persistent homology and hotspot analysis, the spread can be defined for all orthomosaics, which helps to characterise the invasion.

The first step was to cluster blueberry bushes by using specified distances, of which 3 and 6 m were chosen, due to the calculated area of the blueberry bushes. The average diameter was considered to be 2 m for the different sites, and therefore a diameter of 3 m was found to be appropriate to especially group blueberry bushes that were close to each other. The results of both distances were compared and they are listed in Table 2. When blueberry bushes were located in a range of 3 m, they were clustered with the following results. From orthomosaic B3 to B1, 35.51% to 39.87% were clustered. The highest number of clustered bushes were 33 in B3, followed by 25 in B2 and 10 in B1. In comparison to B1 to B3, B4 and B6 had around 28.3 % clusters with three and more blueberry bushes. The highest number within a cluster was nine for B4 and 13 for B6. After increasing the range to 6 m, the number of blueberry bushes clustered in the group 3 or more bushes increased to 69.57%, which is more than 30 percentage points. B1, B3, and B4 had a similar increase of around 15 percentage points and reached 56.63 % in B1, 50.43% in B3 and 44.35 in B6. With less than 10 percentage points, 37.68 % of the blueberry bushes were grouped together with more than three bushes.

**Figure 6:**
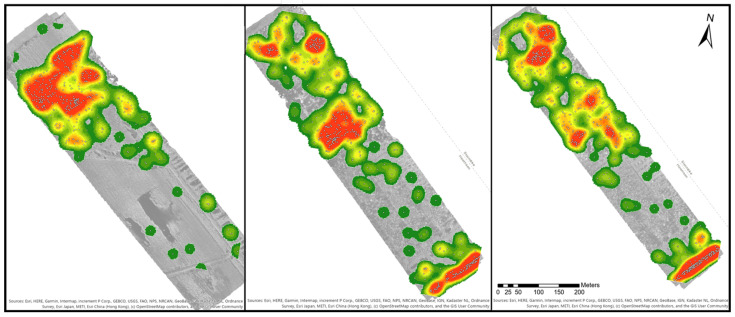
Density map for the blueberry species for the sites B1 to B3 (location see Figure 1). Areas of low densities are marked in green and high densities are red coloured. A gradient between green and red represents values of medium density. White points mark the location of the blueberry bushes.

Based on the point shapefiles of the blueberry bushes, density maps were generated to see how the bushes were distributed within the map. Figure 6 provides three examples of the orthomosaics B1 to B3. Areas with a high density are marked in red and low densities in dark green. Orthomosaic B1 has one large density spot in the northwestern part of the map, while the southeast direction the density decreases with only single or paired bushes. Orthomosaic B2 shows four density spots. Two smaller ones were located in the northwest, a larger spot is in the middle of the orthomosaic and a final one in the southeast. The space between the middle and southeast spot is covered by blueberry bushes, which was similar to the distribution of B3. In orthomosaic B4, nearly the whole area is covered with green to reddish colours. There are three dense spots in the northwest, two spots in the middle and one in the southeast. In comparison to B2 and B3, the spots are smaller. Orthomosaic B6 covers a larger area than all other orthomosaics, but only three density spots could be identified in the middle of the orthomosaic. There were smaller groups of blueberry bushes along the borders of the orthomosaic, and single ones are distributed close to the groups of bushes.

Another analysis focused on the point analysis to generate a map of hotspot areas in order to analyse the spread of the blueberry species. The point analysis used the manually marked blueberry bushes to identify where the proximity of the bushes was significantly different (hot and cold), and to quantify those that were not identified as significantly different. In B1, two 90% confidence hotspots were found in the north. 21 clusters were identified to be 90% significantly different from the study area. The hotspots in B2 were concentrated in the south-easternmost part of the orthomosaic. 26 clusters (out of 220) were significantly different to the study area with a confidence interval of 99%. These points contained all of the bushes located in the south-easternmost part of the orthomosaic. In B3 16 out of the 214 clusters fell into the 99% confidence interval, all located in the south-east of the orthomosaic. The same characteristic was found in B4. 23 clusters out of 248 were found to be significant with a 99% confidence and seven points with 90% to 95% confidence. B4 was the only orthomosaic containing two points considered to be cold spots with 90% confidence in the centre of the orthomosaic.

**Figure 7:**
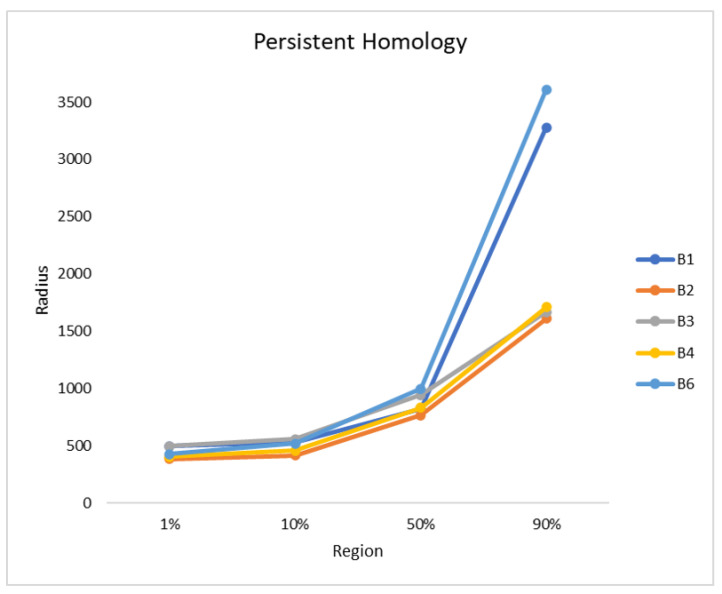
The persistent homology is plotted by radius against the region. Four fused regions were considered: 1%, 10%, 50%, and 90% and all sites are plotted.

Finally, the persistent homology was performed, as described in Section 2.3.1. The radius was plotted against the fused region, as can be seen in Figure 7. The orthomosaics B2, B3, and B4 show a similar trend, while B1 and B6 also follow a different, but similar, trend to each other. B2 is the first orthomosaic, where 1% of the blueberry bushes were fused with a radius of 386 and B1 needed the largest radius with a value of 497. In all orthomosaics the radius needed to fuse up to 10% of the blueberry bushes is similar with values between 415 and 557. There is a small gap of approximately 150 between B3 (945), B6 (998), B1 (824), B2 (768), and B4 (831), when 50% of the blueberry bushes were fused. The radius needed to fuse 90% for B1 and B6 are 3279 and 3611, while, for B2 to B4, it is 1610 to 1709.

### 3.2. Deep Learning Results

In this section, we describe the usefulness of deep learning techniques in order to automatically determine the location of blueberry bushes in our data. As stated in Section 2.5, a widely used network (ResNet50) was chosen and two main aspects were studied: how the data balance affects the final classification results and whether using transfer learning resulted in improved results. Several experiments were presented in a previous study [43]; however, we will only focus on the optimal results for this current study.

Regarding transfer learning, *unfrozen* ImageNet [45] weights were considered to initialise the network. When the model weights are *unfrozen*, all of the layers are normally trained and, thus, all the weights are updated. Regarding the data balance, the blueberry patch loss was weighted eight times that of the soil class, and four times the amount of the other classes. We also performed upsampling of the blueberry class by creating 12 new samples for each patch, as detailed in Section 2.5. Finally, the soil class was downsampled to 50% of its original number of patches.

Because three orthomosaics were available, a leave-one-mosaic-out cross validation approach was applied in order to evaluate the results. One of the orthomosaics was used for training, another for validation and the last one for testing. In order to ensure that all orthomosaics were used at least once for training, validation, and testing, we rotated them accordingly. This section presents the averages results for the TPR and the accuracy results of the three testing stages that correspond to each orthomosaic.

By using the optimal settings that are presented in this section, the model improved from a low TPR value of 63.8% for the blueberry class when no data balancing was applied to a value of 93.39%. In both cases, the overall accuracy for all classes remained similar (98.83% and 98.10%, respectively). Furthermore, it could be observed that unfreezing the weights had a positive effect on the TPR. The best TPR value for the *frozen* weights was 37.12% without data augmentation while maintaining an overall high accuracy value of 98.01%. However, when using high data augmentation with *frozen* weights, the best TPR value of 87.99% was obtained at the expense of a lower overall accuracy value of 75.20%. These results suggest that ImageNet weights can be used for this problem, but they need to be updated (and *unfrozen*) when using data augmentation to focus on blueberry bushes due to the differences in images.

**Figure 8:**
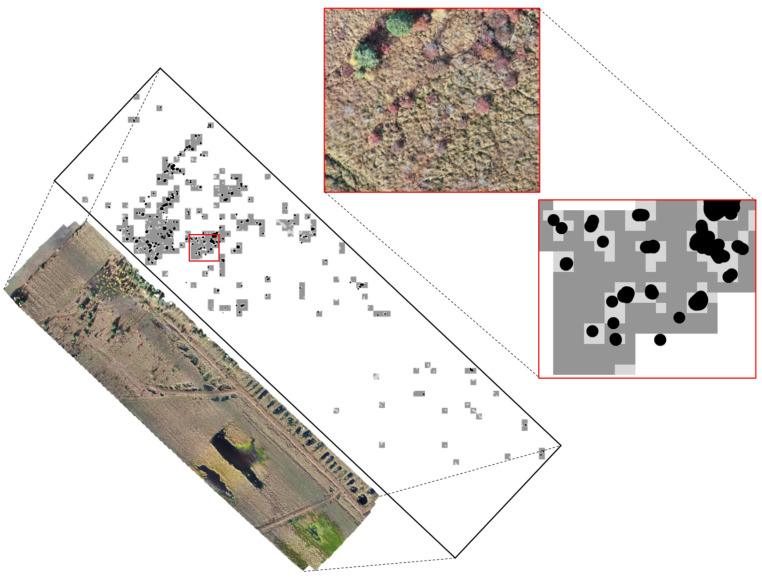
Orthomosaic B1 is displayed with a combination of the manual annotations (black marked spots), the coarse mask (dark grey) and the refined mask (light grey). The red box was zoomed in to show a detailed view on the image and masks.

Regarding the refinement step of the algorithm, Figure 8 presents an example of the obtained results. For most of the predicted patches, the segmentation that was obtained with the refined version of the algorithm is much closer to the manual annotation. However, in a few cases, some of the bushes that had originally been detected are missed after the refinement. Table 3 presents the detailed results of these two issues.

On the one hand, the Dice coefficient, which evaluates the overlap and number of non-GT pixels that are contained in the predicted patches, improved significantly with the refinement algorithm from values around 0.2 to values in the 0.5–0.6 range. On the other hand, the ratio of GT pixels that are covered by the mask, which ranges from 0.88 to 0.95 for the non-refined mask was slightly inferior in the refined version (0.78 to 0.87).

## 4. Discussion

The applied methodology used UAVs to gather information in a restricted access area. Techniques from several research areas were then applied in order to gain knowledge regarding the distribution and properties of the bushes of the invasive blueberry species. In this section, the results that are presented in Section 3 are interpreted in order to assess the stage of the invasion in each of the mosaics.

### 4.1. Difficulties with Data

Blueberry plants show a characteristic red leaf colour in autumn, which make them easily recognisable and identifiable in comparison to other classes of vegetation. Both a simple identification and segmentation by colour were proposed and applied in one of the first segmentation approaches. However, partly visible soil with reddish tones constrained blueberry identification. This problem was especially critical for small blueberry bushes. In autumn, the leaf colours can vary between red, red with a yellowish tone, and partly black. This caused challenges for the annotations and for the deep learning algorithm, since the number of blueberry images was already low in comparison to the other classes and it made the colour approach not usable for this study. Further complications were given by light conditions during image taking. When the blueberry bushes had brighter red colours due to sun light, it was difficult to distinguish them from the ground. Bushes, which were located in the shadows, especially the ones that had a predominately black colour, were barely recognisable.

The analysis presented some difficulties in the calculations of the height and surface area of the blueberry bushes. The main problems to determine bush height are occlusions, due to nearby trees and difficulties due to dense floor covering vegetation. As the cluster analysis shows, the high density of blueberry bushes in some areas and their proximity increased the possibilities that the bushes were partly covered and the whole bush area was not visible, as already pointed out by [10]. Furthermore, bushes were often located close to trees that have canopies that can cover most of a blueberry bush. The areas calculated for the blueberry bushes, exceeding 4–5 m${}^{2}$, indicated that there has to be more than one bush, which was difficult to identify in the images, as well as for calculating the height. Wetland regions, imaged in the orthomosaics are grassland and covered with dense hassocks. Therefore, the soil is often not visible and the ground annotations often represent the height of the hassocks, which resulted in values of 0 m maximum height and even more bushes showed a value of 0 m, when the median height was calculated for smaller bushes, especially in B4 and B6. Therefore, it is assumed that the hassocks can reduce the real height of the bushes by 30 cm. However, the calculated height values exceeded 3 m, which is unusual for the blueberry species that are studied here. There were errors produced when the annotations contained parts of an overlapping tree canopy, which increased the maximum height. The median height was resistant to outlier values. When ground areas were annotated, the median generally decreased the height values, especially for small bushes. Annotations of the ground need to be set carefully, since the wetland was uneven and depressions could increase the height values of the blueberry bushes. Furthermore, the differences between the max. height and median height can be influenced by the structure of the bush canopy. Because of these difficulties, a correlation between the height and area values of blueberry bushes was not considered, but a comparison of the distribution of these values showed that the distribution was similar and the bush area was larger than the height.

Even though these values are estimations, the applied methodology gave a good overview over a large study area, which cannot otherwise be done by extensive field measurements due to wetland protection regulations. Therefore, despite the difficulties, the achieved results emphasised the following discussion of applications and the qualitative use of the introduced methodology.

### 4.2. Application in Landscape Management

The collection of high resolution images and the gathered information can help to map and visualise the findings of this study. This information can be used in order to easily establish management measures against the further invasion of alien species into the wetlands, as pointed out in previous studies [31,35].

The area that was occupied by blueberry bushes was low in terms of the studied area (covering 1 to 1.5 %), which is lower than the identified 3 to 5% in [15]. However, when only the living vegetation was considered, the number of blueberry bushes was found to increase from 8.2 up to 21.1%. These percentages can be considered to be very high, due to the fact that the species is invasive and it does not belong to this sensitive ecosystem. B6 has the largest area and it contains the smallest number of blueberry bushes, with the smallest height and area values measured. Therefore, the invasion seems to be in an early stage and it should be easier to manage. Nevertheless, as shown by persistent homology and the high number of single bushes after clustering, bushes were wide spread, which increases the area where measures against the blueberry bushes need to be considered. The hotspot analysis and density map of B6 indicated that there are some bushes, which are concentrated in a dense spot in the middle of the area and distributed from there homogeneously. These findings allowed for determining that the progress of the blueberry bushes into this site is low.

B1 has a similar distribution, while the number of blueberry bushes per hectare is doubled in comparison to B6. The density map showed high densities in the northern part of the orthomosaic and a gradual decrease of blueberry bushes in the southern direction, which was confirmed by the hotspot analysis. The density of the bushes was higher than in B6, but, as indicated by the persistent homology, the spread was greater. This indicates that the blueberries invaded but did not reach every region of the site. Furthermore, the blueberry bushes in B1 have the largest bush area within all studied sites. The identified bushes maturity suggests that the blueberry species has invaded the area a long time ago. Because the density map provided the information that the blueberry bushes are mainly distributed in the north, there must be conditions in the south, which prevented further spread.

In comparison, B2, B3, and B4 showed a high spread in the southern direction, because blueberry bushes of various size were found everywhere and there were higher concentration areas and several spreading centres. These findings can be confirmed with the persistent homology and density map, indicating a high progress of the invasion. The three orthomosaics seem to have a homogeneous distribution and a gradual change to lower numbers in the southern direction. However, the significant differences that were found by the hotspot analysis indicate that there are conditions that influence the distribution of the blueberry bushes, as in site B1. In addition, all three orthomosaics show an area with a small density of bushes, which can probably be explained by the high water content in the soil correlated with unsuitable conditions regarding plant growth. The invasion of the blueberry species was characterised as such and far advanced for B2 to B4. Only B4 has smaller bushes that cover a smaller area, which suggests that the invasion is less advanced than for B2 and B3. The spread will probably increase there in the following years.

Furthermore, the distribution of the blueberry bushes can be connected to the proximity of trees, especially in B1 to B3, where bushes are mainly found around pine trees and shrubby birches, since birds use these as rest areas and mainly distribute seeds where they rest. An exception is B4, where the trees are located next to depressions that are filled with water, which confirms the unsuitable living conditions for blueberry species. In general, it seems that more blueberry bushes occur when the density of the living vegetation is higher, which can be explained by better living conditions and a better distribution through birds.

To sum up the results and interpretations, it was found that B6 showed an early stage of invasion. B1 shows an advanced stage of invasion with limitations in the south, while B2 to B4 show a critical, advanced stage of invasion, since the blueberry bushes can be found in the whole study site. The methodology used here helped to assess the stages of invasion.

Our study shows a method for helping preserve a sustainable and adaptive conservation of natural ecosystems. Together with further expert interpretations, a deeper understanding of wetland ecosystems can be achieved [30]. The calculated properties, height and area, are indices that can be used for plant growth monitoring [46], and, therefore, they provide useful information for the practical management of wetlands.

### 4.3. Contribution to Invasive Blueberry Studies in Wetlands

Previous studies were conducted to identify areas of blueberry bush invasions. The first studies were done by [9,14] covering up to 12.5 km${}^{2}$ with estimations of blueberry bush coverage areas. Another study focusing on the invasive blueberry species was presented by [15], studying an area of 4.7 ha, which is nine times smaller than the area that was studied in our work. Reference [10] studied an area of 230 ha and characterised the blueberry invasion. The author conducted 11 days of fieldwork in order to capture the information regarding the cover area of blueberry bushes. The study area is less in our work with 63 ha, but, in comparison to the manual fieldwork, image capturing only took half a day. The pre-processing step needed the longest time, about 4 to 5 h per orthomosaic, since manual annotations were done in the beginning of the work. Therefore, the presented workflow of our study reduces the amount of time for gathering information significantly. The characteristics that were provided by [10] are mainly focused on the blueberry bush cover area, which were 16% of the 230 ha. Other characteristics were provided by general statements about average heights and clustering behaviours. Our study provided height and area measurements for each blueberry bush, which has not been done in previous studies. Based on the orthomosaics, further measures, like hot spot analysis, density calculations, and spread measures, could be performed in order to characterise the blueberry invasion in much more detail than previous studies have shown [10,15]. Furthermore, refs. [10,15] stated a high probability of loss of identification during fieldwork due to the density of the bushes in certain areas near former blueberry plantations, which were therefore predominantly investigated. In comparison to the mentioned studies, our work presents an objective grading, a precise cover area and each bush was detected in the captured high-resolution images. This information is essential in order to compare both the invasion and monitoring in different areas.

### 4.4. Automatic Masks Generation

Deep learning techniques were used in order to assess whether DNN can be used to automatically detect the presence of blueberry bushes. Since the manual annotation of the blueberry bushes is the most time-consuming step of this workflow, this has the potential to greatly extend the range of studies. The results in Section 3.2 show that the ResNet50 network succeeded at the classification tasks associated to our problem and that the best results were obtained by re-training the whole network. In this respect, relying on pre-trained weights from ImageNet to solve our problem after minor re-training of the last layer is suboptimal. A dataset must be large enough to re-train the full networks. In our case, this meant using images that were taken from three orthomosaics covering a total of 33 hectares.

At the same time, the results also quantify how a data imbalance may result in a network that classifies most of the patches correctly, wven if the blueberry bushes were mainly misclassified. To address this problem, data augmentation as well as a loss function that took into account the number of pixels for each class were used to influence the weights which helped to reduce bias of the training towards the correct classification of blueberry bushes.

Wetland image classification was performed by [36,37] identifying four and six classes, reaching an overall accuracy of 94.82% and 82.02%. Transfer learning which was only applied in [36] showed the effectiveness of this technique for natural environment studies, when the dataset is large enough. Interestingly, the best result of [37] was obtained when using multi-view images. We will consider this type of images in our future work. A similar approach to detect invasive species was used by [38]. Their approach applied deep learning with data augmentation and transfer learning. Their highest accuracy was 97.6%, which is slightly lower than our overall accuracies of 98.83% and 98.10%. It is important to consider that, in [38], the dataset was mainly composed of images of the invasive species, while, in our case, only 2.64% of the generated patches contained the invasive species. This illustrates the effectiveness of the techniques used in our approach to overcome the imbalance in our dataset.

The results of the automatic classification were used in order to map the invasive species, which is an important first step, whereby a refined segmentation is essential to effectively determine the exact location of plant invasions [35]. The deep learning applications used classified images, and a refined mask provided the blueberry bush locations. The refinement step was necessary in this application, since most of the blueberry bushes are small. Hence, the refined mask offered a more precise localisation of the blueberry bushes. Even though not all bushes were found and some soil areas were misclassified as blueberry bushes, the refined mask could significantly reduce the time of manual annotations and provide maps of the studied area. Increasing the amount of blueberry data can help to optimize the classification accuracy and the localisation of blueberry bushes. This will be considered in our future research.

The generation of automatic annotation masks, as performed here, will allow for large scale studies with a minimum of disturbances in the studied environment. Therefore, UAVs and image analysis provide accurate and cost-effective surveys that are needed when studying invasive species [31]. The used techniques provided more information than that gathered by previous field surveys in wetland areas [10,14].

### 4.5. Limitation and Future Works

The presented work provided a methodology to analyse invasive species from UAV images. Nevertheless, we faced limitations regarding the data that are described in Section 4.1. The autumn season seemed to be a good timing for image taking, but the difficulties regarding the varying colour of the blueberry bushes should be taken into account more carefully. Image taking could be done when the weather is cloudy and not windy, as suggested in previous studies [47,48]. The chosen flight height of 50 m resulted in high-resolution images, which created heavy orthomosaics. The resolution was reduced to 5–7 cm/pixel to be able to use image processing software. This led to difficulties in identifying blueberry bushes and their properties. For the future, different flight settings can be tested, as presented in [49,50], especially smaller flight areas and a reduced flight height can help to increase the resolution and precision of the results of blueberry bush properties.

The use of deep learning proved to be effective for this problem, but also challenging. As in all artificial intelligence approaches, a sufficient number of images that represent the variability of the desired target is necessary in order to train a supervised model that is usable in practice (in the current studies, this amounted to images that corresponded to 33 hectares). Furthermore, with the size of the current blueberry bush data in these images, careful use of data augmentation as well as a dedicated balancing approach was necessary. In order to improve the detection of blueberry bushes, a larger training dataset would likely be needed. For instance, because blueberry bushes are often planted for blueberry production, those fields could offer a good opportunity to collect new images.

Therefore, the detailed mapping within this study can further improve the results of the current status of the blueberry species invasion. Repeated data collection in the same area, as planned, will provide a year-to-year comparison, which will allow for the monitoring and analysis of the ongoing spread in the wetlands. Changes will be easily detectable within the wetlands and the studied blueberry species without disturbances of vulnerable plant and animal species and habitats, as already mentioned by [30,35].

## 5. Conclusions

In this paper, we introduced a multi-disciplinary methodology to quantitatively evaluate the role of plant species in ecosystems, including invasive species. The use of UAVs makes the approach applicable, even in restricted access areas and it increases the total area that can be studied, greatly exceeding the range of existing field studies. We used this methodology to gather information regarding wetland vegetation. Simple and time-saving methods were applied to classify vegetation and provide information about the properties of the invasive blueberry species found in our study site. The distribution of blueberry bushes was analysed in terms of their density, clustering, and spread. The relative importance of blueberries in the wetland was analysed (number of bushes, bush area, and bush height). This information was transformed into location, density, and hotspot maps to provide advanced visualization tools. Deep learning techniques were used in order to automatically detect and segment blueberry bushes, opening the possibility to further extend the range of similar studies.

## Figures and Tables

**Table 1:** Area and counting measures of blueberry bushes detected in the orthomosaics.

	Orthomosaic Area in ha	Number of Blueberry Bushes	Blueberry Bushes per ha	Blueberry Bush Area in m${}^{2}$	Area per Blueberry Bush in m${}^{2}$
B1	11.64	375	32.21	1331.42	3.55	
B2	10.64	687	64.55	1885.51	2.74	
B3	12.47	566	45.40	1470.24	2.60	
B4	12.44	405	32.54	870.33	2.15	
B6	15.14	235	15.53	278.07	1.18	

**Table 2:** Clustering results that are based on point shapefiles of the sites.

	3 m Clustering			6 m Clustering		
	Grouped 3 or More (in %)	Number of Single Bushes	Highest Count in One Group	Grouped 3 or More (in %)	Number of Single Bushes	Highest Count in One Group
B1	39.87	89	10	56.63	18	25
B2	36.82	87	25	69.57	23	50
B3	35.51	98	33	50.43	36	50
B4	28.34	92	9	44.35	39	22
B6	28.28	51	13	37.68	28	21

**Table 3:** Numerical evaluation of the refinement step of the DNN. The rows marked "refined" stand for the algorithm after refinement, while the rows marked "Coarse" correspond to the algorithm without refinement for each of the three studied orthomosaics. The Dice coefficient, as well as the ratio of blueberry pixels in the Ground truth covered by each of the two masks, are presented.

Orthomosaic	Mask Type	Dice	GT Cover
1	Coarse	0.187	0.953
	Refined	0.526	0.860
2	Coarse	0.264	0.949
	Refined	0.624	0.874
3	Coarse	0.223	0.884
	Refined	0.587	0.789

## Data Availability

The data presented in this study are available on request from the corresponding author.
